# News and Miscellany

**Published:** 1881-01

**Authors:** 


### NEWS AND MISCELLANY.

A Manual of Operative Veterinary Surgery is being prepared for the press by Mr. George Fleming.

A Giant Horse.—A horse, foaled in Ohio, has been purchased by Barnum for exhibition. It is seven feet (twenty-two hands) high, and weighs 2,450 pounds.

A Veterinary Society has been formed in Australia, called the Australian Veterinary Society. It has seventeen active and fourteen honorary members.

Regulating Veterinary Practice.—English veterinarians are trying to get a veterinary act passed by Parliament. The object is to put veterinary medicine upon a legal basis, by obliging all veterinarians to pass an official examination before beginning to practice.

A New Disease broke out among the horses at Vermillion, D. T., last fall, which carried them off in about twenty-four hours. It puzzled the skill of the veterinary surgeons to tell what it was, and at last accounts the matter was still a mystery.

Contagiousness of Epizootic Discharges.—A boy named Fred. Palmer, of Elmira, N. Y., lost an eye during the epizootic epidemic, from direct poisoning. It seems that he had wiped his face with a handkerchief which he had used to remove some mucus that his horse had coughed on his coat sleeve.

Charles D. House, D. V. S., sued Leslie C. Bruce and others, proprietors of the *Turf, Field, and Farm*, for damages for alleged libel in calling him a quack in their paper. The case came to trial recently, before Judge Lawrence, of the Supreme Court, and the jury gave the plaintiff a verdict for $1,050 and costs, amounting to $1,214.80. The trial was a complete vindication of the character and professional skill of Dr. House.

Total Number of Horses in the World.—It has been estimated that of the horses in the world, Austria has 1,367,000; Hungary, 2,179,000; France, about 3,000,000; Russia, 21,470,000; Germany, 3,352,000; Great Britain and Ireland, 2,255,000; Turkey, about 1,000,000; the United States 11,000,000; the Argentine Republic, 4,000,000; Canada, 2,634,000; Uruguay, 1,600,000.

THE CENTRAL PARK ZOOLOGICAL GARDEN.—We notice with much satisfaction that in the tax levy for the coming year, the amount of the appropriation for the support of the Zoological department of Central Park has been increased. This action we heartily commend, as this has now come to be regarded as a great educator of the masses as well as a source of pleasure to them. Whatever may have been the criticisms upon the management of other departments of the Park, against this there has not been a complaint in many years.

BLIND MARES FOR BREEDING.—Mr. S. Tam's mare, Clara P., of Fort Wayne, Indiana, gave birth to a filly, which is totally blind. The eye-balls are entirely lacking. I saw the filly a few hours after foaling, and again yesterday. The filly is lively, growing finely, and seems to get around in spite of its serious defect. The dam was originally gifted with seemingly good eyes up to the time she was five years old, when she commenced to have trouble with her eyes, which eventually resulted in her going blind. Clara P. is by Anthony Wayne, her dam a blind mare, quite a trotter, called Crazy Jane. Clara P.'s filly is a catch foal, the sire supposed to be a young bay stallion by Anthony Wayne, owned by Mr. Oliver Hanna. This seems to prove the unwisdom of using blind mares for breeding purposes. The horses in our city and surrounding country have all been troubled with the epizootic.—*Correspondence of Spirit of the Times.*

THE RATION FOR STREET CAR HORSES in this country is now pretty definitely fixed in all our cities. For summer, this ration is half oats and half corn, ground together, sixteen pounds to each horse, with twelve pounds of cut hay. In winter, sixteen pounds of corn meal, with the same amount of hay, forms the ration. Corn meal alone in summer is too heating; but in winter the corn meal seems well adapted to keeping up animal heat and condition, and, being cheaper than oats, is generally adopted in New York City; but in many other cities half oats is used the year round. If these companies would substitute clover hay for timothy, corn meal would make a well-balanced ration. The clover would make up for the deficiency of the corn meal in muscle-sustaining food. Clover is rejected because it is liable to contain dust, which may develop heaves; but this fear is groundless under the plan now adopted of moistening the cut hay and mixing the meal with it. It is fed in a damp condition, and, therefore, no dust can be present to affect the lungs. Clover hay is not properly appreciated as a food for horses. It has a higher value than timothy, and is usually sold $2 to $3 per ton lower in market. Corn is too much used by some car companies. It is a fattening and "heating" food *par excellence*, and not a muscle-builder. —*National Live Stock Journal.*

DEATH OF A VETERINARY SURGEON FROM LOCKJAW.—Dr. Edgar R. Wing, of Needham, Massachusetts, a veterinary surgeon of note, died some weeks ago of lockjaw, after a short illness. He was attending a horse which was afflicted with this disease, and it is supposed that the saliva from the animal came in contact with a wound on his hand, and was the cause of his death. He was considered a young man of much promise. A short time since he opened an office in Newton, and had gained considerable practice there and in surrounding towns. He was twenty-three years of age, and the oldest son of Austin Wing, long a resident of Needham. While attending to business he complained that his jaws felt stiff and sore, and told a friend that he feared he had symptoms of the lockjaw, and was anxious to get home and consult his physician. He grew rapidly worse, and the disease soon terminated fatally.

We have received an interesting letter from the father of the late Dr. Wing, a part of which we give. It will be seen that he does not think there was anything like contagion in the case, in which opinion he is undoubtedly correct.

"He (Dr. Wing) had not been well for some time previous to this. About three weeks previous he had ophthalmia. Had a dry cough (nervous), complaining of a pain in his side, and he applied iodine to the side up to the time of the appearance of tetanus. The muscles of the body, neck, and head were affected; the limbs were not. His neck and jaws were immovable, his back drawn up, and the muscles over his bowels contracted, leaving a space of about three inches for the bowels. Respiration was unchanged, except at the last more labored. Spasms began on Saturday, P. M., six hours after he was confined to the bed. These were of a mild type, and seemed confined to the body. In the first one the heart beat heavily, then subsided until the second, after which it beat heavily to the end. The brain was not affected. I do not believe he took the disease from the horse. There was a slight dry spot on his thumb, resembling a bruise or blister. It was not inflamed."

Pleuro-Pneumonia on Long Island.—Several cases of this disease were discovered by Inspector Rushmore at Hempstead, L. I., about Dec. 15. The animals were on a stock farm. Those diseased were killed, and the rest quarantined.

The National Live Stock Journal has been attacking a much advertised "hog cholera cure," and it has demonstrated very clearly the fraudulent character of the claims for the nostrum. Our contemporary waged a good fight, and comes out ahead, as usual. The *National Live Stock Journal* is the best periodical of its class in this or any country.

Extinction of Pleuro-pneumonia in Holland.—It is asserted that pleuro-pneumonia has been stamped out of Holland entirely. The methods by which this has been done were, isolation, slaughter if necessary, and inoculation. This last means of guarding against pleuro-pneumonia is asserted to have been remarkably successful in Holland, and to have been demonstrated as a measure that should be widely adopted.

The Increased Transportation of Cattle from the East to the West.—The extent to which cattle are transported from the East to dairy districts in the West is hardly realized. Nearly fifty thousand calves, for instance, were sent to Chicago alone in the last half of 1880. There is no surety that some of these cattle have not pleuro-pneumonia, and the danger of their conveying the disease to Western herds is imminent and alarming. It is announced officially that Chicago is already among the diseased centres, though this announcement is (very wisely) qualified. But if pleuro-pneumonia has not gotten West, it inevitably will do so, unless Congress arouses itself to some action.

Foot and Mouth Disease in England.—A correspondent of the *National Live Stock Journal*, writing from England, says that a great damper to the trade has been caused by the recent outbreak of foot and mouth disease. The infection was introduced probably by a cargo of French sheep which had the disease. These animals were slaughtered at once, but the foot and mouth disease began to crop out very soon after this among neighboring cattle. It has since been spreading rapidly. There is nothing which gives such a practical check to stock interests as this disease. It has done more to reduce the number of horned stock in the United Kingdom than pleuro-pneumonia and cattle-plague put together.

Texas Cattle Fever not Epidemic in Texas.—In a paper read before the American Public Health Association, Dec. 7th, 1880, by Dr. Jos. R. Smith, U.S.A., Medical Director for the Department of Texas, the following statements, the result of an extended inquiry, were made regarding the prevalence of Texas Cattle Fever: That no epidemic disease prevails among Texas-bred cattle living and grazing in that State, but that imported cattle, soon after their arrival, are affected by a disease called Texas or Spanish fever, which is very fatal to them alone.

Dr. Smith stated that the deaths among Texas cattle while herding in that State, or while being driven East, were not very frequent, and that they were then generally due to exposure or starvation. The following facts regarding the cattle that die during transit East were given:

The *age* is from two and a half to seven years. The *liver* weighs from five and a half to fifteen pounds, increasing with age; this organ and the gall-bladder are generally healthy. The *spleen* weighs from one and a half to two pounds, and measures from ten to twenty inches in length by four to five in breadth. Various statements regarding the changes found in the viscera are given, but they are too vague to be of any value.

Transit of Cattle in Railway Cars.—At a meeting of the Farmers' Club, in Cooper Union, Dec. 1st, Mr. J. R. S. Van Vleet, an aged agriculturist, read a paper relative to the treatment of cattle in transit from the West, and the sale of diseased meat in this city. Some of his statements were startling, and they created quite a sensation among the members of the club. He said that the health of this great city is imperiled by the treatment of cattle during their long journey from the plains to their Eastern destination. As the trade annually increases, so does the danger to health increase. Droves of cattle driven on foot to St. Louis generally arrive there in good condition. They are then crowded into cars not properly prepared for the transportation. There are no separate stalls, no means to divide the mass, and thus they continue in all their filthy and crowded condition to the end of their journey. To add to the misery of these poor brutes, no provision is made for feeding and watering them while in transit.

In this connection it may be stated that a new cattle-car has been invented, which provides as much as possible for the comfort of animals in transit. It allows of their being fed and watered, and of their getting good fresh air. The invention has the approval of Mr. Bergh and others. It is thought that it will save the six or seven per cent. loss caused by previous modes of transit from West to East.

Appropriations for Experiments Regarding the Diseases of Animals.—The French Chamber of Deputies has appropriated 50,000 francs for M. Pasteur. The money is to enable him to pursue his investigations into the infectious diseases of animals, a work upon which he has now been specially engaged for four years.

The Losses from Hog-Cholera in Illinois during the years 1877-8-9 are shown in the following table:

| | 1877. | 1878. | 1879. |
|---|---|---|---|
| Fat hogs sold, . . . | 2,455,573 | 2,271,493 | 2,543,278 |
| Gross weight, . . . | 618,804,396 | 555,955,097 | 702,102,812 |
| Number died of cholera, . | 1,445,268 | 1,391,422 | 676,738 |
| Total weight died, . . | 106,949,832 | 139,853,508 | 49,326,591 |

The losses in Iowa, Indiana, and several other Western States nearly equal those of Illinois.

Fasting Frogs.—An immense bull-frog which was put in the pathological laboratory at the Columbia Veterinary College, in November, 1879, is still living and lively, having had but two meals in the mean time. When first obtained he was placed in the glass aquarium along with three small frogs. These he at once proceeded to eat up. After this he had no other meal until the succeeding July, when he was given half a pound of beefsteak. This he swallowed at once, after which he looked as nearly the size of an ox as a frog ever did. That meal lasted him till December. At date of writing he appears somewhat emaciated, but could probably stand it another forty days at least.

A National Cattle Commission, and the Exportation of American Cattle.—The annual report of the Secretary of the Treasury for 1880 contains the following facts and recommendations regarding the exportation and importation of cattle:—"In a letter of Feb. 19, 1880, from this department to the Speaker of the House of Representatives, the attention of Congress was called to the prevalence of the disease known as pleuro-pneumonia, or lung plague, in neat cattle, and some recommendations were made as to the proper legislation on the subject. It may be assumed that this disease has never existed in this country west of the Alleghany Mountains, and that it has not for a long time existed in Canada, or in this country near the line of Canada. The exportation of live horned cattle from the United States is very large, and is rapidly increasing, the cattle going mostly to Great Britain. For the eight months ended Aug. 31, 1880, the value of such animals exported was $12,462,837, which is nearly double the value of the exportation for the same period in 1879. By an order of the Privy Council of Great Britian, all American cattle must be slaughtered at the port of arrival within ten days. The effect of this order is to prevent the shipment of any but fat cattle; and it entails great loss as to that class of animals, by compelling the immediate slaughter of such as are injured or become sick upon the voyage, and therefore of little value for food. It also prevents the owners from driving the cattle from the port of importation to a better market, or from keeping them until the market improves. Furthermore, there is a large demand in England for store or stock cattle, to be fed and fattened in that country for its own markets, a demand which this country could supply to an unlimited extent. It is believed that this trade, if unrestricted, might far exceed the trade in fat cattle. The losses and embarrassments by reason of the order for immediate slaughter, are, commercially considered, very great. The British Government, however, is ready to rescind it when it may be done without danger of spreading pleuro-pneumonia in their country through importations from the United States. The question of the rescission of the order has been the subject of official discussion between this Government and the Government of Great Britian, as well as in Parliament. It is believed that whenever Congress makes provision for the extinction or prevention of the disease, or for such security of the great routes of travel from the West to the seaboard as will make it reasonably certain that the cattle shipped from our ports will not carry infection with them, the order of council requiring immediate slaughter will be rescinded. The recommendation that a commission be created, whose duty it shall be to investigate reports of the existence of the disease, and to collect information respecting it, reporting the results to some department for official publication, is renewed. It is further recommended that such commission be authorized to co-operate with State and municipial authorities and corporations and persons engaged in the transportation of neat cattle, and establish regulations for the safe conveyance of such cattle from the interior to the seaboard, and the shipment of them, so that they may not be exposed to the disease; and that such commission, also, may establish such quarantine stations and regulations as may be deemed neccessary to prevent the spread of the disease by importations from abroad. It is believed that the legislation thus indicated, properly executed, will induce the Government of Great Britain to rescind its order for immediate slaughter, and thus promote a very large increase in the exportation of neat cattle from this country. Whether Congress should go further, and undertake the extirpation of the disease in the States where it now exists, is a question of more difficulty, and it is deemed best to leave that part of the subject for independent consideration."

Fasting Horses.—From experiments made in Paris upon horses, the following conclusions were reached, viz.: (1.) "That a horse can hold out for twenty-five days without any solid nourishment, provided it is supplied with sufficient and good drinking water. (2.) A horse can barely hold out five days without water. (3.) If a horse is well fed for ten days, but insufficiently provided with water throughout the same period, it will not outlive the eleventh day. One horse from which water had been entirely withheld for three days drank on the fourth day sixty liters of water within three minutes. A horse which received no solid nourishment for twelve days was nevertheless in a condition on the twelfth day of its fast to draw a load of two hundred and seventy-nine kilos," about five hundred and fifty pounds.

Trichinosis in Spain.—According to a letter from M. Vic (*Ann. de Méd. Vét., Sept.* 1880), veterinary surgeon at Barcelona, and the official communications of the veterinary inspector of abattoirs, eight persons succumbed to the effects of eating infected meat. Twenty-eight in all were known to have ingested trichinosis pork. The parasites were found in the meat and in the muscles of the persons who died, so that the matter of the cause of death was no longer doubtful. Prof. Mauri took occasion to urge the necessity of official supervision by competent microscopists, in order to avoid future calamities. Spain, it would seem, resembles our own country in the absence of this much-needed surveillance.

Suppressing Pleuro-pneumonia.—Pennsylvania has expended thus far nearly $4,500, says the *Boston Cultivator*, in suppressing pleuro-pneumonia, of which sum $2,365 was paid for killing 150 animals infected with the disease. Over 6,000 examinations were made, and the special agent is said to have traveled nearly 11,000 miles while in the performance of his duty. Maryland has been considered the principal source of infection, but as that State has in operation a law for the suppression of the disease similar to that in force in Pennsylvania, it is believed that its spread in that State will be effectually prevented.

Horse Trade in Cincinnati—The business in horses in this market during the past year, as shown by the returns of the Superintendent of the Chamber of Commerce, to be published in his forthcoming report, was the largest ever done here, except during the war, when phenomenal purchases for the army were made. The actual sale of mules and horses aggregate 28,516. The total receipts for the year were 21,733, and the shipments 18,137 head. The average price per head for horses and mules sold here was $72.25. The kind in best supply and most largely sought after was a medium grade of horses, which ranged from $65 to $85 per head, and the better class of strict railroad horses ranging from $85 to $110 per head. The demand for Eastern and foreign markets has been better than at any time since the war.

The Trade in Live Cattle between this country and Europe has so increased as to supersede in extent that in dressed meats. Five years ago the business was regarded simply as an experiment. In 1879 the shipments reached 105,324, of which amount 33,295 were live cattle, and 72,029 dressed carcasses. For 1880, from January up to the first two weeks of August, the shipments were 64,843 live cattle and 53,533 carcasses of beef—a total of 118,376. The trade amounts in money value to from $32,000,000 to $35,000,000 a year. With the removal of the restrictions on American cattle now imposed in Great Britain, the trade will no doubt attain still larger proportions, there being practically no limit to the supply of cattle available for exportation.

Contagious Diseases Among American Cattle.—Mr. G. Fleming, in a letter to the *London Times* on the above subject, says: The United States at present is quite as dangerous, so far as the importation of cattle is concerned, as the most pest-ridden country in Europe. Indeed, I am not certain whether it is not more so; as, through their utter neglect of veterinary medicine, and their independence of each other, the different States have no machinery by which to control, much less suppress, these calamitous disorders, nor uniform action in preventing its extension. Such is not the case in the poorest of the European kingdoms now on the scheduled list. It is true that a number of States are asserted to be free from the lung plague, but we know not—after our experience of such assertions, and knowing now how impossible it is to obtain reliable information—how far they may be well-founded. It is simply nonsense to talk about the small number of diseased animals which reach our shores from the States as evidence that the present restrictions should be removed or relaxed. Do those who talk know the damage that one infected animal may inflict? If they do not, let them read the history of the disease in South Africa or Australia. Some of these people make light of such contagions, and speak of foot-and-mouth disease as a trifling malady, which should not suffice to exclude foreign live stock from our markets. Here, again, they exhibit their ignorance with the history of animal plagues.

Foot-and-mouth disease has proved a more serious scourge to this country than the cattle plague. If the States are to enjoy an unrestricted traffic in food-producing animals with Great Britain, they should find it necessary to produce a sure and certain clean bill of health. The manner in which this is to be achieved is as well known to them as it is to us; but so long as there is a chance of success for the meat consumers' pseudo-friends they will not attempt it. Until they do so, it is to the interest of the nation at large that the present regulations be maintained and rigidly enforced, not only with regard to the United States, but to every cattle exporting country in which there is a case of contagious disease."

Parasites in Flies.—The possibility that flies may be the bearers of contagions has led to some special studies recently regarding a fungous disease which affects the insect, especially at the fall of the year. The diseased fly may generally be found lying against the window, dead, with a zone of white particles about it. On examining it closely, the hairs on the fly will be found covered by minute white globules, similar to those forming the encircling zone. The abdominal segments are separated by distension, and the abdomen appears stuffed by something. On dissecting such a fly in glycerine and water, the cause of the distension will be apparent by the escape of a mass of mycelium threads, with similar spores to those surrounding the fly; almost all the soft tissues have disappeared, nothing being left save the tracheal tubes and the chitine, the fungus having preyed on the soft parts, and probably suspending its growth from lack of moisture. The fungus is called by Colin *empusa muscæ.* It does not seem capable of propagating the disease to other flies. Little is at present known concerning the life history of the fungus. Fungoid diseases affect many insects, notably the silk worm, the wasp, &c.

The appearances above described should not be confounded with those of the laying of eggs by the fly. When the female fly lays its eggs it dies, and the gross appearances might then be mistaken for funoid disease.

